# Unravelling the role of the gut microbiome in antipsychotic-induced weight gain and metabolic dysfunction in humans and rodents: A systematic review

**DOI:** 10.1080/19585969.2026.2637716

**Published:** 2026-03-10

**Authors:** Maximilian Tufvesson-Alm, Louise Walsh, Sinead Pierce, Finola Keohane, Gerard Clarke, Karen O’Connor, John F. Cryan, Harriet Schellekens

**Affiliations:** ^a^ APC Microbiome Ireland, University College Cork, Cork, Ireland; ^b^ Department of Anatomy and Neuroscience, University College Cork, Cork, Ireland; ^c^ RISE Early Intervention in Psychosis service in South Lee, Cork, Ireland; ^d^ Department of Psychiatry and Neurobehavioural Science, University College Cork, Cork, Ireland

**Keywords:** Gut-brain axis, schizophrenia, weight gain, atypical antipsychotics

## Abstract

Second-generation antipsychotics are frequently linked to weight gain and metabolic dysfunction, yet the mechanisms driving these effects remain elusive. The gut microbiome has been proposed as a potential mediator of these adverse outcomes. This study aimed to investigate the role of the gut microbiota in antipsychotic-induced weight gain. A systematic search of PubMed and Embase was conducted. In total, 24 publications were included in this review, including clinical and preclinical observational and intervention studies. Collectively, there is strong evidence that atypical antipsychotic-induced weight gain and metabolic dysfunction is accompanied by microbiota alterations. However, there is a lack of consensus with regards to the exact mechanisms and involvement of the microbiome in antipsychotic-induced weight gain. Nevertheless, a few patterns and common observations were found across studies, such as reduced diversity, increased *Firmicutes/Bacteroidetes* ratio and a reduction in *Akkermansia* species. While microbiota-targeted interventions had generally weak effects on weight gain and metabolic dysfunction in clinical cohorts, the use of specific probiotic strains and microbiota metabolites showed promise in preclinical studies. Thus, while the relationship between antipsychotic-induced weight gain, metabolic dysfunction, and changes in the gut microbiome are evident, further research is warranted to establish definitive causal relationships and to aid in the development of precision microbiota-targeted interventions to counteract these adverse effects.

## Introduction

Antipsychotic medications have evolved through a series of pivotal milestones that have transformed the landscape of psychiatric pharmacotherapy and schizophrenia treatment. The first generation, or typical antipsychotics were noted for their efficacy of treating psychosis due to its strong antagonistic properties on dopamine (Carlsson and Lindqvist 1963). However, these were associated with a high occurrence of disabling adverse effects, including extrapyramidal symptoms, such as parkinsonism and tardive dyskinesia, due to dopaminergic blockade in the nigrostriatal pathway (Marsden and Jenner 1980). The second generation of antipsychotics, or atypical antipsychotics (AAPs), such as clozapine, risperidone and olanzapine, exhibit a broader spectrum of therapeutic effects, addressing not only the positive symptoms of schizophrenia but also the negative symptoms (Abi-Dargham and Laruelle 2005). In this regard, AAPs exhibit less dopamine receptor blockade, have a broader mechanism of action and impact various other neurotransmitter pathways, including serotonergic (Abi-Dargham and Laruelle 2005). While unquestionably transformative in the management of psychiatric conditions, AAPs are accompanied by notable adverse metabolic side effects, including weight gain, diabetes mellitus and hyperlipidaemia (De Hert et al. 2011). Weight gain is a common and significant concern in clinical populations, highlighting the urgent need for a comprehensive understanding of its underlying biological mechanisms (Bak et al. 2014). Current research has implicated both direct and indirect factors contributing to the weight gain associated with atypical antipsychotics. For example, AAPs commonly antagonise a combination of histamine H_1_ receptors and a variety of serotonergic receptors. In this regard, H_1_ antagonism leads to increased appetite, reduced thermogenesis and sedation, resulting in weight gain (Kroeze et al. 2003; Kim et al. 2007; He et al. 2013). Further, antagonism of serotonergic receptors, such as the 5-HT_2C_, has been strongly associated with increased appetite and caloric intake (He et al. 2021). Indirectly, disruptions in central nervous system pathways regulating energy balance and satiety, such as those involving the hunger-regulating peptides leptin and ghrelin may also play a role in observed weight gain (Jin et al. 2008; Panariello et al. 2012). Continual investigation into the specific mechanisms and potential interventions to mitigate these adverse effects is crucial for optimising therapeutic benefit of atypical antipsychotics.

In recent years, there has been growing appreciation for the gut-brain axis and its profound influence on various physiological functions, metabolism and mental health, including schizophrenia (Szeligowski et al. 2020; Liu et al. 2022; Murray et al. 2023). The gut microbiome, comprising of the microorganisms residing in the digestive tract, has emerged as a key player in shaping weight regulation and metabolic health (Fujisaka et al. 2023). Xenobiotics, including psychiatric medications such as AAPs, interacts reciprocally with the microbiome (Clarke et al. 2019; Cussotto et al. 2019a, 2021; Seeman 2021; Minichino et al. 2024). Changes in the microbiota, whether induced by medications or other factors, may contribute to metabolic disturbances and weight gain (Saad and Santos 2025). Despite numerous links established between body weight and the composition of gut microbiota the exact mechanism of these links remains elusive (Boscaini et al. 2022). Similarly, while it appears evident that psychiatric drugs including AAPs can induce alterations in the gut microbiome (Cussotto et al. 2019b; Maier et al. 2018) and that antipsychotic-induced weight gain is strongly influenced by the microbiome, the exact mechanism of this interaction is unknown (Davey et al. 2012, 2013; Bretler et al. 2019; Vasileva et al. 2022; Dias et al. 2024). Understanding the interplay between the gut microbiome and host metabolism holds promise for providing novel insights into the mechanisms underlying antipsychotic-induced weight gain and facilitate the development of targeted interventions to mitigate these adverse effects (Dinan and Cryan 2018; Gorbovskaya et al. 2020). Therefore, investigating the gut microbiome represents a promising avenue for improving the metabolic health among antipsychotic medication recipients. The gut microbiota’s involvement in nutrient absorption, energy metabolism and inflammation regulation positions it as a potential modulator of antipsychotic-induced weight gain (Boscaini et al. 2022; Fujisaka et al. 2023; Soliman et al. 2023; Saad and Santos 2025). Nonetheless, the specific mechanisms through which the gut microbiome influences these processes remain unclear, presenting a dynamic area of ongoing research. This systematic review seeks to synthesise existing evidence, shedding light on the complex interplay between AAPs and the gut microbiome, and the potential of microbiota-targeted interventions to reduce antipsychotic-induced weight gain. Furthermore, it aims to identify gaps in current knowledge and propose potential avenues for future investigation, offering valuable insights into strategies for mitigating the adverse effects associated with these widely prescribed antipsychotic medications.

## Material and methods

### Search strategy and selection criteria

The PubMed and Embase were used to find relevant publications. All publications underwent screening for inclusion in accordance with the criteria outlined in Table 1. Given the limited available information on this topic, both preclinical and clinical studies were considered for inclusion. Publications must have included adult rodents or humans; children and adolescents were excluded and considered a confounding factor which limits the general interpretation due to biological and aetiological differences in the context of antipsychotic-induced weight gain, and its potential effect on interventions. Thus, the study of antipsychotic-induced weight gain and microbiota changes in children and adolescents falls outside the scope of this review and should be subject to a separate review. Further, atypical antipsychotic medication was deemed necessary for treatment, with the acceptance of using any other medications concurrently. A measure or classification of weight or metabolic health was also needed to assess changes due to antipsychotic-induced weight gain. Inclusion of a follow up microbiota or biometric analysis was necessary to meet inclusion criteria, so that results could be analysed and potentially correlated to any microbiome alterations observed. A systematic search of PubMed and Embase was conducted with the following search terms: (microbiota OR microbiome) AND (antipsycho*) AND (weight OR metabolic OR adipos* OR ‘BMI’ OR ‘body mass index’) AND (human OR rodent OR mice OR mouse OR rodent OR rat). The full inclusion and exclusion criteria are outlined in Table 1. The review commenced with an initial title screening phase (07/09/2025), during which titles were either accepted for further screening or excluded from consideration. Following this, the remaining papers underwent abstract screening, where the abstracts were carefully reviewed to determine if they met the inclusion criteria for advancement to the full text screening phase. Publications meeting the criteria were then subjected to full text examination, and those meeting the inclusion criteria were included in the review. The primary searches, subsequent screenings and data extractions were done by two independent reviewers (M.T-A. and L.W.). A full list of all screened articles and the reason for their exclusion can be found in Supplementary Table 1.

**Table 1.** Inclusion and exclusion criteria.

Inclusion criteria	Exclusion criteria
Adult rodents or humans of either sex	Studies in children and adolescents
Atypical antipsychotic with any other interventions	Studies of first-generation antipsychotics
Microbiota analysis or intervention	Primary focus on metabolism of antipsychotics
Both preclinical and clinical studies assessing metabolic health (BMI, weight etc.,)	Publications not in English or full text not available

### Data extraction

Selected publications were categorised by clinical or preclinical studies and observational or interventional studies. Predefined data measures were extracted, including weight gain, metabolic function measures, microbiota analysis measures, as well as methodological information, such as participant information, treatment details and intervention procedure.

### Assessments

This review strived to follow the PRISMA guidelines (Page et al. 2021). As such, a Cochrane risk-of-bias assessment was conducted on included publications (Sterne et al. 2019). The results were visualised using robvis (McGuinness and Higgins 2021) and can be found in Supplementary Figure 1-2. In general, some concerns were raised regarding the risk-of-bias, mainly stemming from lack of, or lack of reporting, on blinding during data collection and analysis. Further, a certainty assessment was done in accordance to the GRADE guidelines (Schünemann et al. 2008) and can be found in Supplementary Table 2. Generally, a moderate certainty level was observed. The main factors lowering the certainty levels were due to some concerns from risk-of-bias assessment and imprecision due to low effect sizes. This was in particular seen in studies with a relatively short duration (i.e., a few weeks).

## Results

### Publication selection

**Figure 1.**
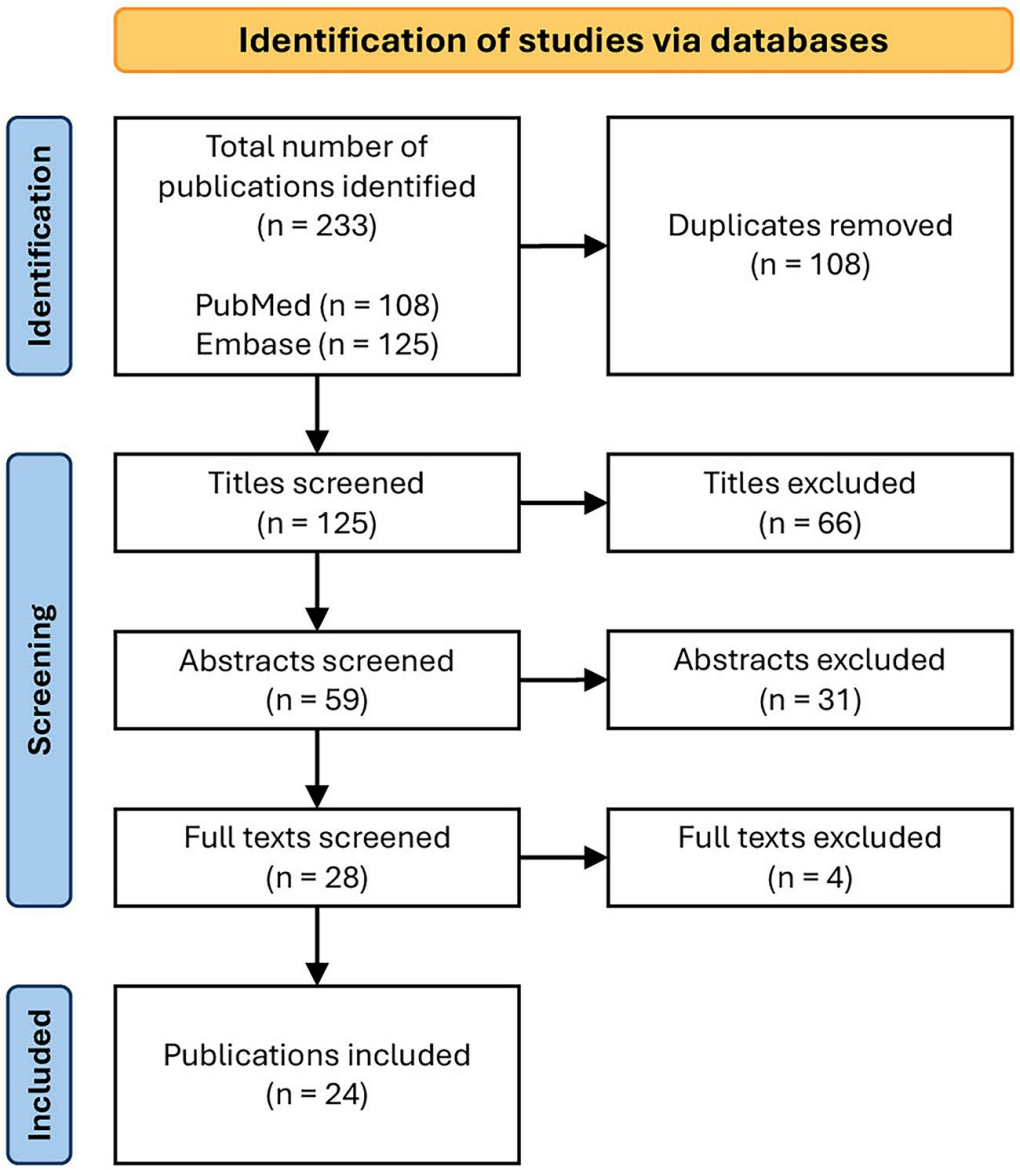
Flow diagram outlining the selection process.

The initial search returned a total of 229 results, 102 duplications were deleted, and the remaining 127 titles were screened. Subsequently, 59 titles were accepted for abstract screening. From these, 28 publications were selected for full-text screening. The screening process is visually represented in Figure 1. In total, 24 studies were included in this review.

### Study characteristics and microbiota measures

This systematic review accumulates 24 studies, with publication dates ranging from 2013 to 2025. The selected publications were divided into clinical (*n* = 10) and preclinical studies (*n* = 14), and further into observational (*n* = 6 and *n* = 6 for clinical and preclinical studies, respectively), and intervention studies (*n* = 4 and *n* = 8 for clinical and preclinical studies, respectively).

The reporting of microbiota measurements varied strongly across studies. The most consistently reported measure was the *Firmicutes* and *Bacteroidetes* either as a ratio (F/B ratio) or individually. In studies where the ratio was not specifically provided, the F/B ratio change was estimated where simultaneous and opposite changes to F and B was observed.

### Observational evidence of weight gain and metabolic dysfunction with atypical antipsychotic treatment

In clinical observational studies, weight gain and BMI increase was consistently associated with AAP treatment in both bipolar disorder (BD), first-episode psychosis (FEP) and schizophrenia (SCZ) patients, where reported (Table 2). Here, only one observational study considered sex differences and reported that weight gain was only observed in females following six weeks of olanzapine (OLZ) treatment (Pełka-Wysiecka et al. 2019). Of note, this study had a relatively short treatment period compared to the other clinical studies, which may impact the strength of the observations. However, Yang et al. similarly observed increased weight gain in females compared to males (Table 3). Two clinical study reported on measures of metabolic health (Huang et al. 2022b; Yuan et al. 2018) and observed an increase in fasting blood glucose, low density lipoprotein, triglyceride levels, and insulin resistance index (IRI) associated with AAP treatment.

**Table 2.** Observational clinical studies.

Author, Year	Subjects and treatment	Weight and BMI difference	Metabolic health measures	Microbiota changes
Flowers et al. 2017	BD patients on AAP (*n* = 49) or other treatment (*n* = 68).	↑ BMI with AAP treatment.	Not reported.	↓ α-diversity in female AAP. = α-diversity in male AAP. ↑ Lachnospiraceae, ↓ Akkermansia, ↓ Sutterella with APP treatment.
Yuan et al. 2018	FEP patients treated with RIS (*n* = 41) and followed for 24 weeks.	↑ Weight and ↑ BMI with treatment.	Fasting ↑ Glucose, ↑ IRI, ↑ LDL and ↑ TG with treatment.	↑ Bifidobacterium spp., ↑ E. Coli, ↓ Clostridium coccoides, ↓ Lactobacillus spp. with treatment.
Pełka-Wysiecka et al. 2019	SCZ patients (*n* = 20) given OLZ and followed for 6 weeks.	↑ Weight with treatment in females only.	Not reported.	No significant effects seen.
Yin et al. 2023	Long-term SCZ patients on clozapine divided in those with metabolic syndrome (*n* = 28) and without (*n* = 33).	N/A.	N/A.	↓ α-diversity in metabolic syndrome vs. without. ↓ Faecalibacterium in males with metabolic syndrome vs. without. ↑ Escherichia/Shigella in females with metabolic syndrome vs. without.
Liu et al. 2024	SCZ patients treated with high metabolic risk APs that developed obesity (*n* = 49) or not (*n* = 98) looked at retrospectively, 1-2 years from start of treatment.	↑ BMI in both groups over time.	Not reported.	↓ Bacteroides, ↓ Parabacteroides, ↓ Akkermansia, and ↓ Clostridium in obese patients vs. non-obese.
Zhao et al. 2024	Long-term SCZ patients divided into those with metabolic syndrome (*n* = 63), high risk (*n* = 88) or no metabolic syndrome (*n* = 22), based on BMI, diabetes and fasting glucose levels.	N/A.	N/A.	↓ α-diversity in metabolic syndrome vs. none. ↓ Senegalimassilia in metabolic syndrome and high risk patients vs. none.

Clinical studies investigating the metabolic effect of atypical antipsychotics (AAP) in the context of the microbiome. ↑: Significant increase, ↓: Significant decrease, =: No significant change, compared to its respective control group. SCZ: Schizophrenia, BD: Bipolar disorder, FEP: First-episode psychosis, AAP: atypical antipsychotics, OLZ: Olanzapine, RIS: Risperidone, IRI: Insulin resistance index, LDL: Low density lipoprotein, TG: Triglycerides.

**Table 3.** Clinical intervention studies targeting the microbiome to treat AAP-induced weight gain.

Author, Year	Subjects and treatment	Weight and BMI difference	Metabolic health measures	Microbiota changes
Yang et al. 2021	SCZ and SCZA patients under OLZ treatment given probiotics (*n* = 34) or placebo (*n* = 33) for 12 weeks.	↑ Weight with OLZ overall and significantly more in women compared to men. ↓ Weight gain with probiotics at 4 weeks.	Not reported.	Not reported.
Huang et al. 2022a	Study 1: FEP patients treated with OLZ given probiotics (*n* = 39) or placebo (*n* = 37) for 12 weeks. Study 2: FEP patients treated with OLZ given probiotics and dietary fibre (*n* = 30) or placebo (*n* = 28) for 12 weeks.	= Weight and = BMI with probiotics vs. placebo. ↓ Weight and ↓ BMI with probiotics + dietary fibre vs. placebo.	Fasting ↓ Insulin, ↓ IRI and = glucose with probiotics vs. placebo. Fasting ↓ Insulin, ↓ IRI and ↓ HDL with probiotics + dietary fibre vs. placebo.	Not reported.
Huang et al. 2022b	SCZ patients under AAP treatment given probiotics + dietary fibre (*n* = 32), probiotics (*n* = 29), dietary fibre (*n* = 29) or placebo (*n* = 28) for 12 weeks.	↑ Weight and ↑ BMI in placebo-group. ↓ Weight and ↓ BMI gain significantly reduced compared to placebo with all treatment.	Fasting ↑ Insulin and ↑ IRI in placebo-group. Fasting ↓ Insulin, ↓ IRI and ↓ glucose with all treatments vs. placebo.	↑ α-diversity and ↓ F/B ratio with probiotics + dietary fibre only compared to placebo. Microbiota richness was associated with weight-loss.
O’Donnell et al. 2022	HC (*n* = 22), FEP (*n* = 5) and established SCZ patients (*n* = 17) on AAP underwent lifestyle intervention (diet and exercise) for 12 weeks.	= Weight and = BMI with intervention.	Not reported.	= α-diversity, ↑ Proteobacteria ↑ Firmicutes phyla, ↓ Lactobacillus, ↓ Fusobacteria, ↓ Ruminococcos and ↓ Copracoccus in SCZ vs. HC. ↑ α-diversity with intervention in SCZ.

↑: Significant increase, ↓: Significant decrease, =: No significant change, compared to its respective control group. SCZ: Schizophrenia, SCZA: Schizoaffective disorder, FEP: First-episode psychosis, HC: Healthy controls, AAP: atypical antipsychotics, OLZ: Olanzapine, IRI: Insulin resistance index, HDL: High density lipoprotein. Probiotics were a mix of different probiotic strains unless otherwise specified.

Further evidence can be found in preclinical studies (Tables 4 and 5). Weight gain and increased white adipose tissue was a consistent finding with olanzapine (OLZ) and risperidone (RIS) treatment, with few exceptions. Interestingly, Huang et al. (2021) showed that OLZ only increases weight gain when on a normal chow diet, whereas when on a high-fat diet (HFD) there was no effect of OLZ on weight gain. This is in line with other studies utilising HF or high-fat high-sugar (HFHS) diets showing no or reduced weight and body fat with AAP treatment (Abolghasemi et al. 2021; Chen et al. 2025). Another interesting finding is that, where investigated, lower dose olanzapine seems to have stronger effect on weight gain than higher (Davey et al. 2012; Qian et al. 2023). Similarly, Zeng et al.reported reduced weight gain when treating with a relatively high dose, 8 mg/kg, whereas most studies used 2–4 mg/kg. Since the first (Davey et al. 2012) showed that antipsychotic-induced weight gain was only present in female rats, subsequent studies have commonly used female subjects to study antipsychotic-induced weight gain. Of the included preclinical studies, nine used female rodents, three used male and two included both. With the exception of one study (Abolghasemi et al. 2021), female rodents consistently exhibited clear antipsychotic-induced weight gain. Conversely, in studies including males only two out of five showed clear antipsychotic-induced weight gain. Although it should be mentioned that these also included HFD and higher dose of OLZ.

**Table 4.** Observational preclinical studies.

Author, Year	Subjects and treatment	Weight change	Other metabolic health measures	Microbiota changes
Davey et al. 2012	Male and female SD rats (*n* = 8 per group) received OLZ (2 or 4 mg/kg IP) or vehicle for 3 weeks.	↑ Weight in females with OLZ. Lower dose having larger effect. = Weight in males. ↑ Visceral fat overall with OLZ.	↑ Food intake and ↓ locomotion with OLZ. ↓ Blood ghrelin with OLZ in females. ↑ GHSR expression with OLZ in males.	= α-diversity. ↑ F/B ratio with OLZ
Morgan et al. 2014	Female c57Bl6 mice (*n* = 8–12 per group) on HFD received OLZ (∼5 mg/kg in food) for 10 weeks.	↑ Weight gain with OLZ.	Not reported.	= α-diversity. ↑ Erysipelotrichi with OLZ and associated with weight gain.
Bahr et al. 2015	Female c57Bl6 mice (*n* = 5 per group) received RIS (80 ng or 80 µg/day in drinking water) or vehicle for 58 days.	↑ Weight gain with higher dose RIS vs. vehicle.	↓ Resting metabolic rate with higher dose RIS vs. vehicle.	↓ β-diversity and ↑ F/B ratio with RIS vs. baseline and vehicle.
Abolghasemi et al. 2021	Female c57Bl6 mice (*n* = 8 per group) on HFHSD received OLZ (2–4 mg/kg by oral gavage) for 9 weeks.	↓ Weight gain and ↓ body fat with OLZ.	↓ Blood glucose and ↓ IRI with OLZ.	= α-diversity and = F/B ratio with OLZ.
Qian et al. 2023	Female SD rats (*n* = 11 per group) received different doses OLZ (2, 4 or 8 mg/kg in drinking water) for 4 weeks.	↑ Weight gain with OLZ. More prevalent at lower doses.	↑ Blood TG with the low dose OLZ only.	↓ β-diversity and ↑ F/B ratio with OLZ.
Kamath et al. 2024	Male and female SD rats (*n* = 4 per group) received OLZ or Lurasidone (both 7.5 mg/kg by oral gavage) for 3 weeks.	↑ Weight gain with OLZ. = Weight gain with Lurasidone	↑ Blood glucose and ↑ TG with OLZ. = Blood glucose and = TG with Lurasidone	↓ α-diversity and ↑ F/B ratio with OLZ. ↑ α-diversity with Lurasidone.

Preclinical studies investigating the metabolic effect of atypical antipsychotics (AAP) in the context of the microbiome. ↑: Significant increase, ↓: Significant decrease, =: No significant change, compared to its respective control group. HFD: High-fat diet, OLZ: Olanzapine, RIS: Risperidone, GHSR: Growth hormone secretagogue receptor, IRI: Insulin resistance index, TG: Triglycerides.

**Table 5.** Preclinical studies utilising microbiota-targeted interventions to prevent antipsychotic-induced weight gain.

Author, Year	Subjects and treatment	Weight change	Other metabolic health measures	Microbiota changes
Davey et al. 2013	Female SD rats (*n* = 9-10 per group) received OLZ (2 mg/kg IP) or vehicle for 3 weeks combined with ABX.	↑ Weight gain and ↑ periuterine fat with OLZ. ↓ Weight gain and ↓ periuterine fat with ABX vs. OLZ.	↑ Food intake, ↑ Plasma FFA, ↓ IRI ↓ locomotion with OLZ. ↓ Plasma FFA with ABX vs. OLZ.	↑ F/B ratio with OLZ. ↓ F/B ratio with ABX vs. OLZ.
Kao et al. 2018	Female SD rats (*n* = 6 per group) received OLZ (10 mg/kg IP) or vehicle for 2 weeks combined with prebiotic (B-GOS).	↑ Weight gain with OLZ. ↓ Weight gain with prebiotic vs. OLZ.	Not reported.	No significant changes with OLZ or prebiotic + OLZ.
Huang et al. 2021	Female c57Bl6 mice (*n* = 10 per group) on control or HFD received OLZ (5 mg/kg by oral gavage) together with probiotic (*A. muciniphila*) for 16 weeks.	↑ Weight gain with OLZ on control diet. = Weight gain with OLZ on HFD. ↓ Weight gain with probiotic vs. OLZ.	↓ Locomotion with OLZ. ↑ Locomotion with probiotic vs. OLZ.	Not reported.
Zeng et al. 2024	Male c57Bl6 mice (*n* = 8 per group) received OLZ (8 mg/kg IP) combined with prebiotic (B-GOS) for 8 weeks.	↓ Weight gain with OLZ. = Weight gain with prebiotics vs. OLZ.	↑ Blood TC and ↑ TG with OLZ. ↓ Plasma TC and ↓ TG with prebiotic vs. OLZ.	↓ α-diversity and ↓ *Akkermansia* with OLZ. ↑ α-diversity and ↑ *Akkermansia* with prebiotics vs. OLZ.
Mushraf et al. 2024	Male Wistar rats (*n* = 6 per group) received OLZ (2 mg/kg IP) together with probiotics for 90 days.	↑ Weight gain with OLZ. ↓ Weight gain with probiotic vs. OLZ.	↑ Blood pressure, ↑ TC, ↑ TG and ↓ HDL with OLZ. ↓ Blood pressure, ↓ TC, ↓ TG and ↑ HDL with probiotic vs. OLZ.	↑ F/B ratio with OLZ. ↓ F/B ratio with probiotic vs. OLZ.
Chen et al. 2025	Male c57Bl6 mice (*n* = 9 per group) on HFD received OLZ (20 mg/kg IP) combined with probiotic (*A. muciniphila*) for 8 weeks.	= Weight gain with OLZ. ↓ Weight gain with probiotic vs. OLZ.	↑ Blood TC, ↑ TG and ↑ insulin with OLZ. ↓ Blood TC, ↓ TG and ↓ insulin with probiotic vs. OLZ.	↓ *Akkermansia* with OLZ. ↑ α-diversity with probiotic vs. OLZ.
Aboulalazm et al. 2025	Female c57Bl6 mice (*n* = 9-10 per group) received RIS (∼40 µg/kg in drinking water) combined with probiotic (*L. reuteri*) or the metabolite reutericyclin for 9 weeks.	↑ Weight gain and ↑ perigonadal fat with RIS. ↓ Perigonadal fat with probiotic vs. RIS. ↓ Weight gain with both probiotic and reutericyclin vs. RIS.	↑ Feeding efficiency with RIS. ↓ Feeding efficiency with both probiotic and reutericyclin vs. RIS.	Distinct shift in microbiome with all treatments. No restoration.
Hadiono et al. 2025	Female c57Bl6 mice (*n* = 12 per group) received RIS (∼40 µg/kg in drinking water) combined with the metabolite reutericyclin for 5 weeks.	↑ Weight gain with RIS. ↓ Weight gain with reutericyclin vs. RIS.	↑ Feeding efficiency, ↓ locomotion and ↓ anaerobic metabolism with RIS. ↓ Feeding efficiency, ↑ locomotion and ↑ anaerobic metabolism with reutericyclin vs. RIS.	↑ β-diversity with reutericyclin vs. RIS.

↑: Significant increase, ↓: Significant decrease, =: No significant change, compared to its respective control group. ABX: Antibiotic cocktail, HFD: High-fat diet, HFHSD: High-fat high-sugar diet, OLZ: Olanzapine, RIS: Risperidone, IRI: Insulin resistance index, LDL: Low density lipoprotein, HDL: High density lipoprotein, TC: Total cholesterol, TG: Triglycerides, FFA: Free fatty acids. Probiotics were a mix of different probiotic strains unless otherwise specified.

Despite these observed differences in antipsychotic-induced weight gain, other metabolic health measures were consistent across dose and diet. In this regard, increased food intake, reduced locomotion and metabolic rate, and increased blood markers, such as glucose and triglycerides were repeatedly associated with AAPs, with the exception of one publication (Abolghasemi et al. 2021). Only one preclinical study compared AAPs other than OLZ and RIS, and found that lurasidone, generally considered to have a comparably lower risk of weight gain, did not induce weight gain or metabolic changes.

### Observational evidence of microbiota changes with atypical antipsychotics

The earlier preclinical studies investigating antipsychotic-induced weight gain provide evidence of the involvement of the microbiome. In this regard, microbiota depletion was shown to attenuate OLZ-induced weight gain (Davey et al. 2013). Building on this, a subsequent study utilising germ-free mice show that the microbiome is both necessary and sufficient for antipsychotic-induced weight gain (Morgan et al. 2014).

This was further evident in clinical studies. Overall, a reduction in α-diversity was associated with APP treatment and metabolic syndrome, along with changes to various bacterial genre (Table 2). Of these, *Akkermansia* was found in more than one study to be associated with APP and antipsychotic-induced weight gain (Flowers et al. 2017; Liu et al. 2024). Notably, Flowers et al. observed α-diversity changes in females, but not in males, in line with other studies showing a larger effect of antipsychotic-induced weight gain in female subject. Further, Pełka-Wysiecka et al. did not observe any microbiota changes following their relatively short treatment period of 6 weeks.

In preclinical studies, AAPs was associated with significant changes in the microbiome composition (Tables 4 and 5). However, changes to α-diversity were not consistently observed where three studies reported no changes and two reported lower α-diversity following AAP treatment. Additionally, two studies reported reduced β-diversity. These changes were accompanied by several other microbiota changes. In particular, an increased F/B ratio was reported in six studies, and two studies reported reduced *Akkermansia* abundance following AAP treatment. Finally, lurasidone was associated with increased α-diversity (Kamath et al. 2024).

### Clinical interventional studies targeting the microbiome to treat antipsychotic-induced weight gain

Four clinical studies were included that utilised interventions with the aim to treat antipsychotic-induced weight gain through the microbiota. A direct approach using a mix of probiotic strains with or without dietary fibre supplement was the most common, whereas one study utilised lifestyle intervention, including dietary and exercise intervention, aimed to alter the microbiota. Collectively, probiotics alone had weak effects on antipsychotic-induced weight gain, with only one study reporting significantly reduced weight gain (Huang et al. 2022b), one study reporting reduced weight gain only at the 4-week timepoint over a 12-week study (Yang et al. 2021), and one study reporting no effect at all (Huang et al. 2022a). However, where reported, probiotics where able to reduce fasting blood insulin and IRI, and one of two studies reported reduced blood glucose with probiotics compared to placebo (Huang et al. 2022b, 2022a). The combination of probiotics and dietary fibre were investigated in two studies and proved more effective than probiotics alone, and led to significantly reduced weight gain, BMI and metabolic health measures, including fasting blood glucose, insulin, HDL and IRI. Further, the combination of probiotics and dietary fibre induced changes in the microbiome, including increased α-diversity and reduced F/B ratio, which was not observed with probiotics alone (Huang et al. 2022b). They further reported that microbiota richness was associated with weight-loss. Finally, lifestyle intervention did not alter weight or BMI but caused an increase in α-diversity (O’Donnell et al. 2022).

### Preclinical interventional studies targeting the microbiome to treat antipsychotic-induced weight gain

To complement the clinical intervention studies, eight preclinical studies using microbiota-targeted interventions were included. Of these, four used probiotics (*A. muciniphila, L. reuteri* or mix of several strains), two utilised prebiotics (B-GOS), two investigated the bacterial metabolite reutericyclin and one used antibiotic cocktail (ABX). Probiotics was consistently reported to reduce antipsychotic-induced weight gain as well as improve other associated metabolic parameters, including blood pressure, TC, TG and insulin. Further, *A. muciniphila* increased locomotion (Huang et al. 2021) and *L. reuteri* reduced feeding efficiency (Aboulalazm et al. 2025). Further, the probiotic mix and *A. muciniphila*, reduced F/B ratio and increased α-diversity in AAP-treated animals, respectively.

Prebiotics however had mixed results. In Kao et al. B-GOS was able to reduce weight gain caused by OLZ, but did not report any other metabolic changes and saw no significant microbiome-related changes, either with OLZ alone or with the addition of B-GOS. Notably, this 2-week paradigm was relatively short compared to other preclinical studies. In the other prebiotic study, no weight gain effect was seen, either with OLZ alone or with the addition of B-GOS over 8 weeks (Zeng et al. 2024). However, B-GOS reduced OLZ-induced increases in blood TC and TG, as well as increased the observed reduction in α-diversity and *Akkermansia* abundance caused by OLZ.

Following the positive data regarding *L. reuteri*, two recent studies investigated the effect of a metabolite of *L. reuteri*, reutericyclin (Aboulalazm et al. 2025; Hadiono et al. 2025). Both studies found that reutericyclin reduced weight gain induced by RIS, and increased locomotion and reduced feeding efficiency and anaerobic metabolism. These changes were accompanied by a distinct shift in the microbiome, including increased β-diversity.

Lastly, Davey et al. observed that ABX treatment reduced OLZ-induced weight gain and blood free fatty acid (FFA) levels, as well as restored the F/B ratio in the microbiome.

## Discussion

This review provides an overview of recent research exploring the relationship between the gut microbiome, antipsychotic-induced weight gain, and metabolic dysfunction in both humans and rodents. The collective findings emphasise the impact of AAP treatment on metabolic health. Overall, there is strong evidence to support a role of the microbiome in antipsychotic-induced weight gain. However, there is a lack of consistent pattern and clear consensus regarding the precise nature of the alterations or the role of the gut microbiome in antipsychotic-induced weight gain and associated metabolic dysfunction. Nonetheless, there is persuasive evidence of compositional shifts in the gut microbiome following antipsychotic treatment, with some consistency across the findings.

Differences in research design are notable, considering both preclinical and clinical studies were included. The treatment length varied drastically across the included studies, ranging from a few weeks to long-term treatment. Further, the clinical studies included here used a variety of AAPs at different doses, which were not always specified in detail. Contrarily, preclinical studies used mainly OLZ, with only three using RIS, and one investigating lurasidone as a control. However, a diverse range of doses and administration routes were used. All the above-mentioned parameters can strongly impact the results, potentially explaining some of the variability.

Nonetheless, across the various experimental models and clinical cohorts in this review, consistent findings highlight that significant weight gain and metabolic dysfunction often accompany AAP treatment. Notably, this effect was more pronounced in female participants, who also tended to exhibit greater metabolic dysfunction compared to males, correlating with existing literature (Seeman 2009; Seguin et al. 2023; Ercis et al. 2024). Such observations remain consistent across both preclinical and clinical studies. Although few of the included clinical studies reported on sex differences, those that did confirm greater antipsychotic-induced weight gain seen in females (Pełka-Wysiecka et al. 2019; Yang et al. 2021), as well as greater microbiota changes following AAP treatment (Flowers et al. 2017). In agreement, the first preclinical study included here reported weight gain associated with OLZ in females only (Davey et al. 2012). Consequently, the use of only female subjects in preclinical studies were more common, accounting for nine of the fourteen included studies, whereas three used only males and two included both. Subsequent preclinical studies further confirm that antipsychotic-induced weight gain is more pronounced in females. However, factors such as AAP dose and diet appear to also impact antipsychotic-induced weight gain. For example, the few included studies comparing different doses showed that a lower dose OLZ resulted in greater weight gain (Davey et al. 2012; Qian et al. 2023). In line with this, two studies using higher doses OLZ (≥8 mg/kg) showed no or even reduced weight gain (Zeng et al. 2024; Chen et al. 2025). This observation might be related to side-effects of OLZ that is more prevalent at higher doses, such as nausea and stomach discomfort, which could potentially reduce appetite in rodents. Furthermore, a higher dose of AAPs might also reduce food reward and reward-related eating through blocking dopaminergic reward pathways, thus reducing excessive eating and weight gain. Similarly, this may help explain the discrepancy between antipsychotic-induced weight gain with animals receiving a regular chow diet and HF or HFHS diets, which is more dependent on reward-related feeding. Indeed, antipsychotic-induced weight gain has previously been shown to correlate to striatal reward activity (Nielsen et al. 2016), although this interaction appears complex (Fell et al. 2008). Nevertheless, these discrepancies collectively indicate an elaborate interaction between AAPs and weight gain.

To understand these mechanisms, the studies included in this review offer distinct insights into the underpinnings of antipsychotic-induced weight gain. Multiple hypotheses have been proposed to explain this phenomenon, reflecting the intricate interplay between antipsychotics and metabolic processes. For example, Davey et al. (2012) observed a reduction in circulating ghrelin and increased hypothalamic GHSR expression, suggesting a link through the ghrelinergic system, which plays a key role in hunger and feeding control. Notably, ghrelin acts through GHSR-heterodimer complexes with receptors also commonly targeted by AAPs, such as the 5-HT_2C_, known to directly affect hunger (Schellekens et al. 2015; Xu et al. 2017; He et al. 2021). Thus, ghrelin and AAPs could directly interact with each other to affect hunger and feeding behaviour. However, the observed changes suggest a reduction in hunger and are consistent with observations in obesity, likely as a compensatory mechanism for advanced weight (Gupta et al. 2021). Thus, it is difficult to pinpoint causality as these changes may instead be a consequence of weight gain.

A consistent finding in preclinical studies was that AAP treatment was associated with reduced locomotion (Davey et al. 2012; Huang et al. 2021; Hadiono et al. 2025), reduced metabolic rate (Bahr et al. 2015), reduced anaerobic metabolism and increased feeding efficiency (Aboulalazm et al. 2025; Hadiono et al. 2025), i.e., increased conversion of calories into weight gain. Collectively, this suggests that AAPs reduce energy expenditure, resulting in weight gain. This is not surprising considering known effects of AAPs, including sedation through strong histaminergic inhibition and reduced locomotion through dopaminergic blockade.

Furthermore, three preclinical studies investigated inflammation, and found that OLZ treatment was associated with increased inflammation (Huang et al. 2021), and specifically elevated TNF-α (Kao et al. 2018; Kamath et al. 2024). Interestingly, inflammation and increased TNF-α in particular is strongly associated with obesity and the development of metabolic disease, including diabetes (Sethi and Hotamisligil 2021). Additionally, OLZ was associated with elevated TNF-α in the jejunum (Kamath et al. 2024), suggesting that AAPs induces inflammation in the gut and can directly impact and alter the microbiome. This is further strengthened by evidence that AAPs, including both OLZ and RIS, inhibit commensal bacteria growth (Morgan et al. 2014; Bahr et al. 2015; McDonagh et al. 2025). Interestingly, Davey et al. (2013) was able to show that microbiota depletion through ABX effectively prevented antipsychotic-induced weight gain. This finding was further expanded on in subsequent studies, showing that germ-free mice do not exhibit antipsychotic-induced weight gain (Morgan et al. 2014) and that faecal microbiota transfer from RIS-treated mice to controls induced changes in metabolism similar to RIS (Bahr et al. 2015). Taken together, these findings show that the microbiome could have a causal role in antipsychotic-induced weight gain.

In line with this, both clinical and preclinical studies consistently showed that alterations to the microbiome were associated with antipsychotic-induced weight gain, with few exceptions. However, the different methodologies and variations in study design, combined with the vastness and diversity of the microbiome, make it challenging to elucidate the exact role of the microbiota in these processes. Nevertheless, a few consistent patterns could be identified. Firstly, AAP treatment and antipsychotic-induced weight gain was overall associated with a reduction in microbiota diversity, consistent with the observation that AAPs inhibit growth of commensal bacteria (Morgan et al. 2014; Bahr et al. 2015). Secondly, while AAP induced diverse changes to microbial abundance at a phylum and family level, certain patterns could be identified. For instance, an increase in *Firmicutes* prevalence, a decrease in *Bacteroidetes* levels and an increased ratio between these (F/B ratio) was associated with AAP and antipsychotic-induced weight gain overall.

An elevated F/B ratio has previously been associated with obesity (Koliada et al. 2017; Palmas et al. 2021). However, the F/B ratio is a broad measure, and its relevance has been contested due to its low reproducibility and weak evidence (Sze and Schloss 2016; Walker and Hoyles 2023; Chanda and De 2024). Thus, it is difficult to interpret the relevance of the observed changes in F/B ratio in this review, and likely the interaction between the microbiome and antipsychotic-induced weight gain is much more complex and requires more specific measures.

Here, a few bacterial genera were identified in several studies. As such, a reduction in *Akkermansia* (Flowers et al. 2017; Liu et al. 2024; Zeng et al. 2024; Chen et al. 2025) and *Lactobacillus* (Yuan et al. 2018; O’Donnell et al. 2022) abundance was associated with AAP treatment and weight gain. In line with this, Akkermansia has previously been characterised as particularly sensitive to AAPs (Maier et al. 2018). Interestingly, species from both of these have been described to protect against metabolic disease and weight gain, in particular *A. muciniphila* and *L. reuteri*. (Cani et al. 2022; Aboulalazm et al. 2025; Ma et al. 2025).

Considering the observed alterations and importance of the microbiome to mediate antipsychotic-induced weight gain, the microbiota emerges as a natural target for intervention to treat antipsychotic-induced weight gain. Preclinical intervention studies using prebiotics were limited but overall showed beneficial effects on OLZ-induced weight gain (Kao et al. 2018; Zeng et al. 2024). Stronger evidence was found for probiotics. Thus, in line with previous studies and observations regarding *Akkermansia* and *Lactobacillus*, probiotic preparations of *A. muciniphila* or *L. reuteri* had strong beneficial therapeutic effect against antipsychotic-induced weight gain and associated metabolic measures and inflammation (Huang et al. 2021; Aboulalazm et al. 2025; Chen et al. 2025). In an effort to further elucidate the mechanisms underlying these effects, it was demonstrated that the addition of reutericyclin, a metabolite of *L. reuteri*, was sufficient for therapeutic effect (Aboulalazm et al. 2025; Hadiono et al. 2025).

Instead of a specific strain, a mix of several probiotic strains was continually used in clinical interventions. While a probiotic mix had beneficial effects in mice (Mushraf et al. 2024), the effects were less pronounced in humans (Yang et al. 2021; Huang et al. 2022a, 2022b). However, the number of studies was limited. The best therapeutic effect on antipsychotic-induced weight gain and associated metabolic health measures appears to be a combination of both probiotics and dietary fibre (Huang et al. 2022b), in line with previous studies (Soliman et al. 2023). Nevertheless, these studies highlight the limits of current microbiota-targeted interventions and the difficulties in translating their effects from animals to a clinical setting.

While an involvement of the microbiome in antipsychotic-induced weight gain appears evident, many questions regarding this interaction remains unanswered. Specifically, the mechanisms by which the microbiome contributes to weight gain following AAP treatment and to what extent remains unclear. A major limitation in primarily the preclinical studies included in this review is the lack of diversity in antipsychotics used. Mainly OLZ was used with a few studies using RIS to induce weight gain. However, there are several other antipsychotics that are associated with weight gain, metabolic dysfunction and microbiota changes and this represents a major gap in the field currently. Importantly, by investigating a diverse range of antipsychotics, common factors affecting the microbiome and metabolic health can potentially be identified. This knowledge would be crucial to develop novel antipsychotics without, or at least with reduced negative effects on the microbiome and metabolic health. While microbiota-targeted interventions represent a promising approach to treat antipsychotic-induced weight gain, there is also a need for novel antipsychotic treatment strategies without these adverse. Furthermore, novel formulation approaches such as engineered gut microbiota-targeting microcapsules can improve bioavailability of antipsychotic drugs while simultaneously be beneficial to the microbiome through the use of material that facilitates fermentation, such as inulin (Meola et al. 2024). Thus, there are several avenues to improve antipsychotic-induced metabolic dysfunction and gut dysbiosis, including microbiota-targeted interventions, the use of novel gut-neutral antipsychotics and novel formulation approaches that mitigates negative effects.

The recognition of gender-specific disparities in both microbial composition and metabolic outcomes, as well as the diverse results observed in the different studies, underscores the necessity for personalised approaches. Such approaches should account for individual variations in both metabolic and microbiota responses to antipsychotic medications (Wang et al. 2025). Future research into characterising metabolic and microbiota signatures associated with antipsychotic-induced weight gain are warranted and could provide valuable insights into potential targets for therapeutic interventions aimed at mitigating metabolic side effects associated with antipsychotic treatment (Kaddurah-Daouk and Weinshilboum 2014; Gokulakrishnan et al. 2023; Cheng et al. 2024; Ioannou et al. 2024). Additionally, longitudinal studies examining changes in microbial composition and function over the course of antipsychotic treatment are essential. Such investigations could elucidate temporal patterns of microbial dysbiosis and its association with metabolic outcomes, ultimately informing the development of more effective strategies for managing metabolic side effects in individuals with schizophrenia and related disorders (Bastiaanssen and Cryan 2021). Further exploration into the use of microbiota-targeted interventions for antipsychotic-induced weight gain is imperative. Studies in rodents have demonstrated promising effects in mitigating weight gain, highlighting the potential efficacy. However, there is a need for in-depth investigation into the underlying mechanism of action to translate these findings into human models effectively. If successful, probiotic treatments could emerge as potential adjunct therapies, complementing standard AAP treatment protocols.

In conclusion, this review underscores the complex relationship between antipsychotic treatment, gut microbiome, and metabolic health. While the interaction between antipsychotic-induced weight gain and the microbiome is evident, the lack of consensus on the microbiome’s role highlights the need for further research. Nevertheless, evidence to date suggests that microbiota-targeted interventions may provide a promising avenue for offsetting metabolic dysfunction and weight gain associated with antipsychotic treatment.

## Supplementary Material

Supplementary Material.pdf

Supplementary Table 1.xlsx
