# Supplementary material for: Unravelling the role of the gut microbiome in antipsychotic-induced weight gain and metabolic dysfunction in humans and rodents: A systematic review
Source: Dialogues Clin Neurosci. 2026 Mar 10;28(1):131–44. doi: 10.1080/19585969.2026.2637716 (PMC12978182; doi:10.1080/19585969.2026.2637716)
Supplement: Supplementary Material.pdf [file TDCN_A_2637716_SM1225.pdf]

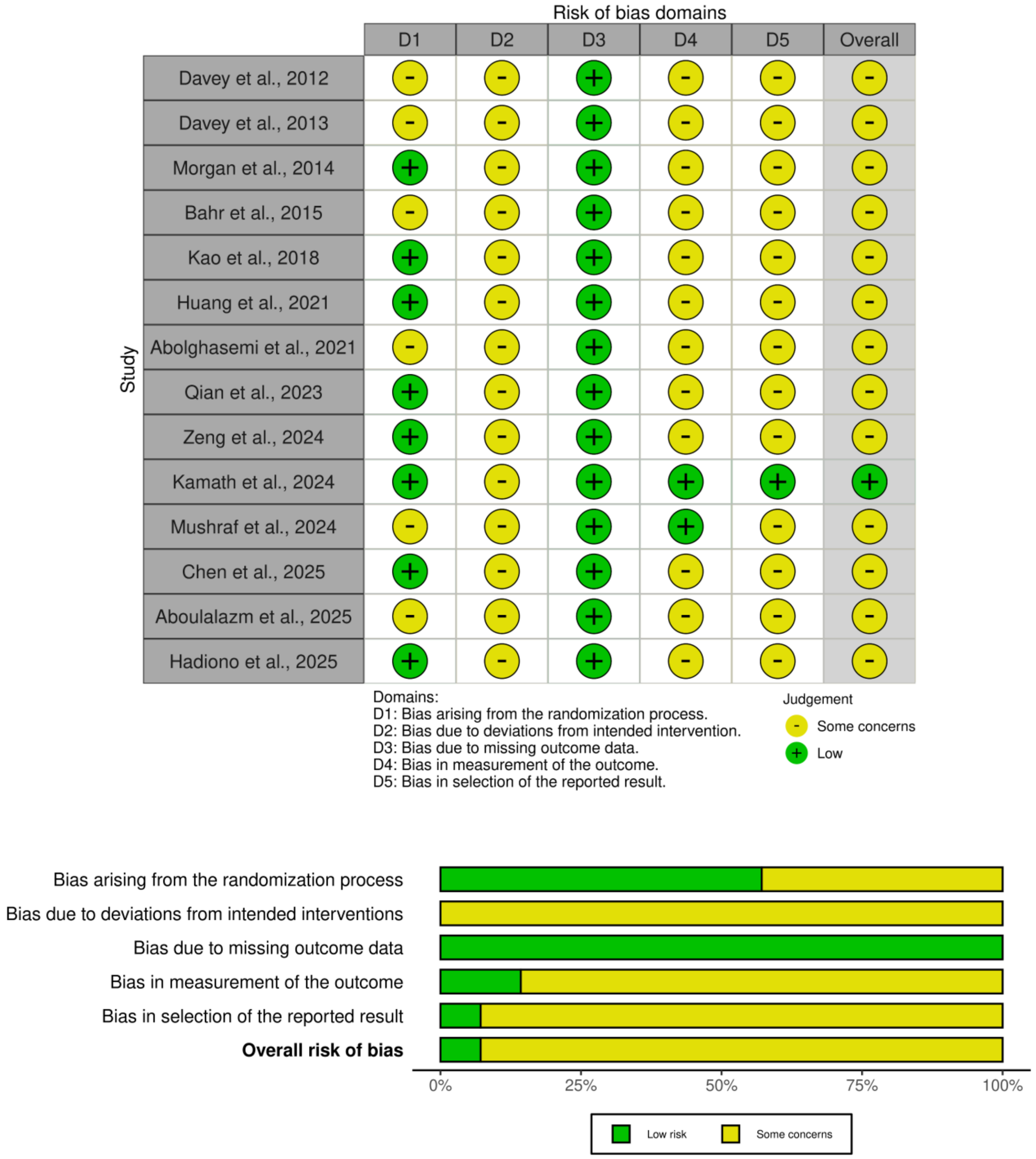

Supplementary Figure 1. Risk of bias assessment of preclinical studies.

Risk-of-bias assessments were done on all included preclinical studies using Cochrane risk-of-bias tool and summarized in the figure above. Figure was made using *robvis*.

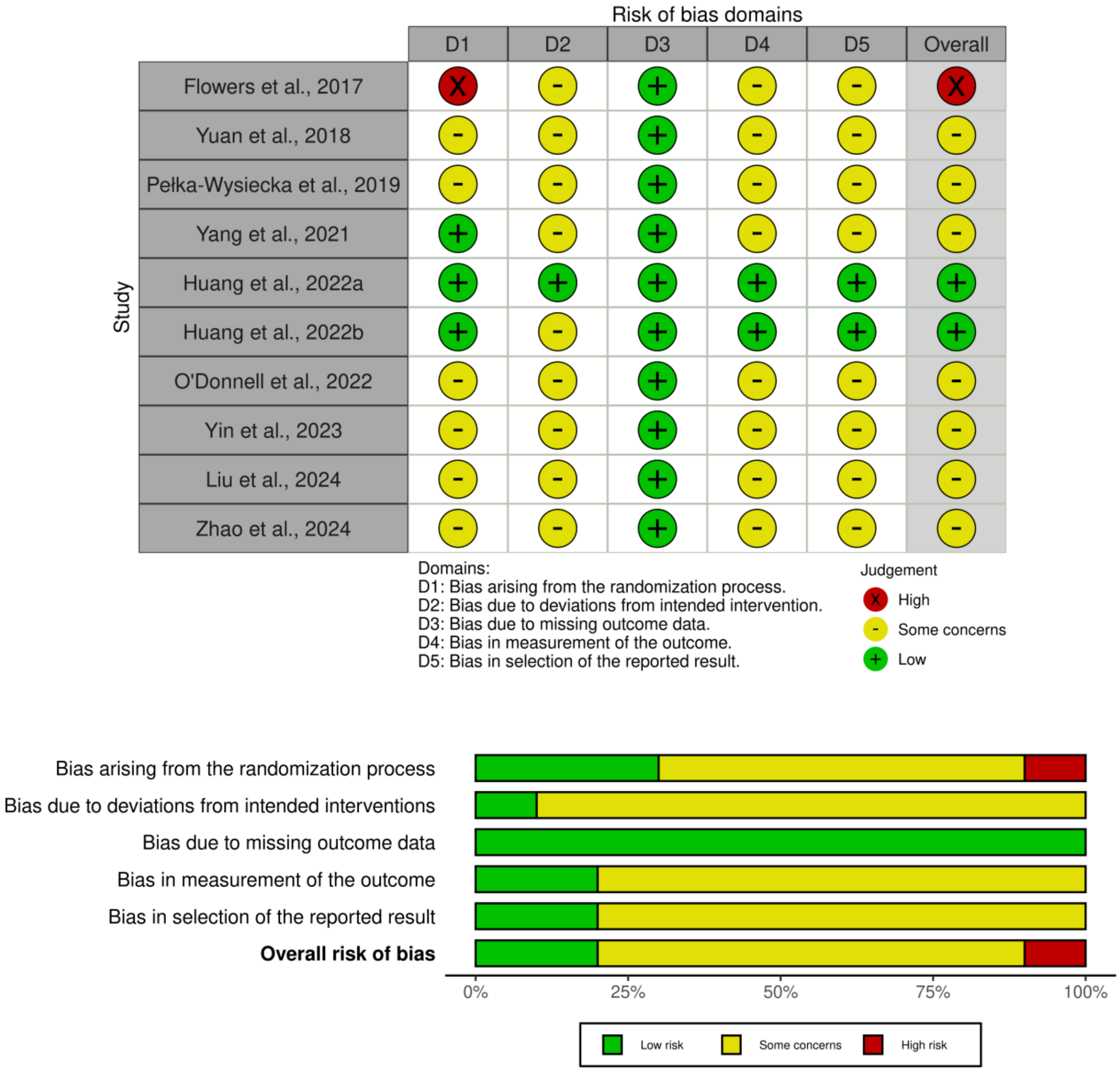

Supplementary Figure 2. Risk of bias assessment of clinical studies. Risk-of-bias assessments were done on all included clinical studies using Cochrane risk-of-bias tool and summarized in the figure above. Figure was made using *robvis*.

	Study	Study design	Risk of bias	Inconsistency	Indirectness	Imprecision	Certainty
Preclinical	Davey et al., 2013	Intervention – Non-randomized (-1)	Some concern (-1)	No concern	No concern	No concern	**Low**
	Kao et al., 2018	Intervention – Randomized	Minor concerns	No concern	No concern	Some concern (-1)	**Moderate**
	Huang et al., 2021	Intervention – Randomized	Minor concerns	No concern	No concern	Some concern (-1)	**Moderate**
	Zeng et al., 2024	Intervention – Randomized	Minor concerns	No concern	No concern	Some concern (-1)	**Moderate**
	Mushraf et al., 2024	Intervention – Non-randomized (-1)	Minor concerns	No concern	No concern	No concern	**Moderate**
	Chen et al., 2025	Intervention - Randomized	Minor concerns	No concern	No concern	Some concern (-1)	**Moderate**
	Aboulalazm et al., 2025	Intervention – Non-randomized (-1)	Some concern (-1)	No concern	No concern	No concern	**Moderate**
	Hadiono et al., 2025	Intervention - Randomized	Minor concerns	No concern	No concern	No concern	**High**
	Davey et al., 2012	Observational	Some concern (-1)	No concern	No concern	No concern	**Moderate**
	Morgan et al., 2014	Observational	Minor concerns	No concern	No concern	No concern	**High**
	Bahr et al., 2015	Observational	Some concern (-1)	No concern	No concern	No concern	**Moderate**
	Abolghasemi et al., 2021	Observational	Some concern (-1)	No concern	No concern	No concern	**Moderate**
	Qian et al., 2023	Observational	Minor concerns	No concern	No concern	No concern	**High**
	Kamath et al., 2024	Observational	Low risk	No concern	No concern	No concern	**High**
Clinical	Yang et al., 2021	Intervention - Randomized	Minor concerns	No concern	No concern	Some concern (-1)	**Moderate**
	Huang et al., 2022a	Intervention - Randomized	Low risk	No concern	No concern	No concern	**High**
	Huang et al., 2022b	Intervention - Randomized	Low risk	No concern	No concern	Some concern (-1)	**Moderate**
	O'Donnell et al., 2022	Intervention – Non-randomized (-1)	Some concern (-1)	No concern	No concern	Some concern (-1)	**Very low**
	Flowers et al., 2017	Observational	High risk (-2)	No concern	No concern	No concern	**Low**
	Yuan et al., 2018	Observational	Some concern (-1)	No concern	No concern	No concern	**Moderate**
	Pełka-Wysiecka et al., 2019	Observational	Some concern (-1)	No concern	No concern	Some concern (-1)	**Low**
	Yin et al., 2023	Observational	Some concern (-1)	No concern	No concern	Some concern (-1)	**Low**
	Liu et al., 2024	Observational	Some concern (-1)	No concern	No concern	No concern	**Moderate**
	Zhao et al., 2024	Observational	Some concern (-1)	No concern	No concern	No concern	**Moderate**

Supplementary Table 2. Certainty assessment.
Certainty assessments were done on all included studies according to the GRADE guidelines and summarized in the table.
